# ERβ Regulation of Gonadotropin Responses during Folliculogenesis

**DOI:** 10.3390/ijms221910348

**Published:** 2021-09-26

**Authors:** Eun B. Lee, V. Praveen Chakravarthi, Michael W. Wolfe, M. A. Karim Rumi

**Affiliations:** 1 Department of Pathology and Laboratory Medicine, University of Kansas Medical Center, Kansas City, KS 66160, USA; elee10@kumc.edu (E.B.L.); praghavulu@kumc.edu (V.P.C.); 2 Department of Molecular and Integrative Physiology, University of Kansas Medical Center, Kansas City, KS 66160, USA; mwolfe2@kumc.edu; 3 Institute for Reproduction and Perinatal Research, University of Kansas Medical Center, Kansas City, KS 66160, USA

**Keywords:** estrogen receptor β, follicle stimulating hormone, luteinizing hormone, steroidogenesis, follicle development, oocyte maturation, ovulation

## Abstract

Gonadotropins are essential for regulating ovarian development, steroidogenesis, and gametogenesis. While follicle stimulating hormone (FSH) promotes the development of ovarian follicles, luteinizing hormone (LH) regulates preovulatory maturation of oocytes, ovulation, and formation of corpus luteum. Cognate receptors of FSH and LH are G-protein coupled receptors that predominantly signal through cAMP-dependent and cAMP-independent mechanisms that activate protein kinases. Subsequent vital steps in response to gonadotropins are mediated through activation or inhibition of transcription factors required for follicular gene expression. Estrogen receptors, classical ligand-activated transcriptional regulators, play crucial roles in regulating gonadotropin secretion from the hypothalamic–pituitary axis as well as gonadotropin function in the target organs. In this review, we discuss the role of estrogen receptor β (ERβ) regulating gonadotropin response during folliculogenesis. Ovarian follicles in Erβ knockout (Erβ^KO^) mutant female mice and rats cannot develop beyond the antral state, lack oocyte maturation, and fail to ovulate. Theca cells (TCs) in ovarian follicles express LH receptor, whereas granulosa cells (GCs) express both FSH receptor (FSHR) and LH receptor (LHCGR). As oocytes do not express the gonadotropin receptors, the somatic cells play a crucial role during gonadotropin induced oocyte maturation. Somatic cells also express high levels of estrogen receptors; while TCs express ERα and are involved in steroidogenesis, GCs express ERβ and are involved in both steroidogenesis and folliculogenesis. GCs are the primary site of ERβ-regulated gene expression. We observed that a subset of gonadotropin-induced genes in GCs, which are essential for ovarian follicle development, oocyte maturation and ovulation, are dependent on ERβ. Thus, ERβ plays a vital role in regulating the gonadotropin responses in ovary.

## 1. Introduction

Follicle stimulating hormone (FSH) and luteinizing hormone (LH) are called gonadotropins due to their effects on gonadal development and function [1,2] Gonadotropins are secreted from the anterior pituitary gland and act on the ovary and testis [1,2]. In the ovary, gonadotropins interact with intraovarian factors to regulate steroidogenesis, follicle development, oocyte maturation, ovulation, and formation of the corpus luteum [1,2,3,4,5] Estrogens synthesized in the ovary during folliculogenesis in turn act on the hypothalamic–pituitary (H–P) axis to regulate gonadotropin secretion [2]. While estrogens generally exert a negative regulatory effect on gonadotropin secretion, a high level of estrogens during the preovulatory period induces a surge of gonadotropins, which is essential for oocyte maturation and induction of ovulation. 

Ovarian follicles consist of oocytes surrounded by two types of somatic cells, granulosa cells (GCs) and theca cells (TCs). These somatic cells are involved in steroidogenesis, and regulation of oocyte development from the dormant stage to ovulation. While TCs are mainly involved in steroidogenesis, GCs are responsible for steroidogenesis, as well as regulation of oocyte maturation. The gonadotropin receptors, FSH receptor (FSHR) and LH/chorionic gonadotropin (CG) receptor (LHCGR), are expressed on the somatic cells, but not on the oocytes. Thus, gonadotropin response that leads to oocyte maturation is mediated through the signaling within somatic cells [6]. 

Estrogen signaling not only regulates the gonadotropin secretion, but it also controls the gonadotropin functions in the ovary [7]. Estrogen receptors are abundantly expressed in the H–P axis as well as in somatic cells in the ovary. While TCs express estrogen receptor α (ERα), GCs cells express estrogen receptor β (ERβ) [8]. ERβ is the predominant estrogen receptor in the ovary, and the adult ovary is the site associated with the highest level of ERβ expression in females [9]. Thus, it is highly likely that ERβ plays a crucial role in regulating ovarian functions, including those mediated by gonadotropins. Loss of ERβ is associated with a decreased estrogen level, and attenuated preovulatory gonadotropin surge associated with complete failure of ovulation [10,11,12,13,14]. In this review, we discuss the role of ERβ in regulating the gonadotropin responses in ovaries. 

## 2. Estrogen Regulation of Gonadotropin Secretion 

**Figure 1:**
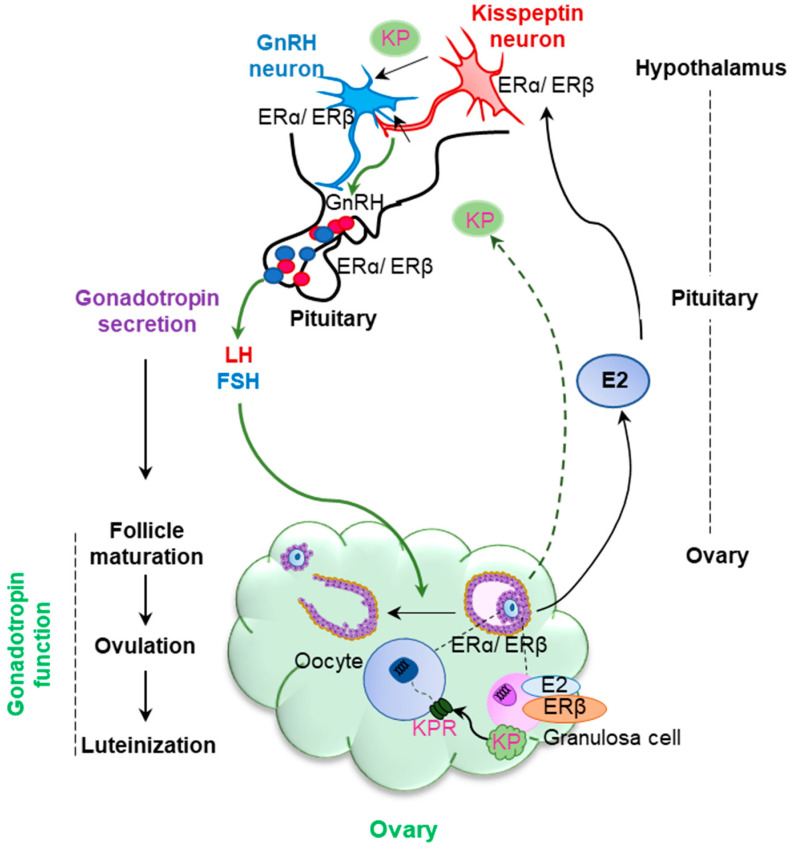
**Estrogen receptor β (ERβ) regulation of gonadotropin production and function.** Estradiol (E2) secreted from ovarian follicles acts on the kisspeptin (KP) neurons in the hypothalamus to regulate KP expression and release. KP acts on GnRH neurons to induce GnRH release in the hypothalamic–pituitary (H–P) axis. GnRH stimulates the gonadotrophs in the anterior pituitary to induce gonadotropin (FSH and LH) secretion. Gonadotropins act on the ovary to induce follicle development, oocyte maturation, ovulation, and luteinization. Estrogen receptors ERα and ERβ are expressed in hypothalamic neurons, as well as in gonadotrophs. While ERα plays a predominant role in KP neurons, ERβ regulates GnRH release and secretion of gonadotropins. Moreover, ERβ is the major estrogen receptor in ovarian follicles. Thus, ERβ plays a vital role in the levels of gonadotropin production and gonadotropin function.

Estrogen signaling plays an essential role throughout the hypothalamic–pituitary–ovarian (H–P–O) axis. Estrogens are synthesized in the ovary during folliculogenesis and circulating estrogens acts on the kisspeptin neurons in the hypothalamus to regulate kisspeptin production. Kisspeptins stimulate gonadotropin releasing hormone (GnRH) neurons in the hypothalamus leading to the secretion of GnRH [15] (Figure 1). Finally, GnRH acts on the gonadotrophs in the anterior pituitary and induces gonadotropin synthesis and release. 

There are two distinct populations of kisspeptin neurons in the hypothalamus; one that is repressed by estrogens and a second that is induced by estrogens [16,17,18,19,20,21]. Kisspeptin neurons in the arcuate nucleus are repressed by estrogen signaling. These neurons are responsible for basal secretion of gonadotropins, which are essential for steroidogenesis, and development of ovarian follicles [16,17,18,19,20,21,22,23]. Throughout the estrous cycle, low levels of ovarian-derived estradiol inhibit GnRH secretion via negative feedback on kisspeptin neurons until the proestrus evening, when elevated estradiol induces a preovulatory GnRH surge [21,24,25,26]. In rodents, kisspeptin neurons in the anteroventral periventricular nuclei and neighboring paraventricular nuclei mediate estrogen induced positive feedback on LH surge [16,17,18,19,20,21]. This high level of estrogen during the preovulatory period that induces the gonadotropin surge is required for oocyte maturation and ovulation. 

Estrogen receptors are expressed in the kisspeptin neurons, GnRH neurons in the hypothalamus as well as in the pituitary gonadotrophs [22]. Estrogen mediated repression of kisspeptin neurons in the arcuate nucleus is mediated by ERα [23]. In the absence of ERα in ERα^KO^ mice and rats, kisspeptin secretion is increased due to the lack of the repressive effects [27,28,29]. An elevated level of kisspeptin results in augmented GnRH release in the hypothalamus, which leads to an increased secretion of gonadotropins from the anterior pituitary [27,28,29]. Ultimately, a high level of gonadotropins acting on the ovaries synthesize an increased amount of estrogens. A high level of gonadotropins associated with elevated levels of estrogens lead to acyclic anovulation and infertility [27,28,29].

In ERβ^KO^ mice and rats, steroidogenesis and follicle maturation are significantly reduced, which is associated with an attenuated gonadotropin surge [10,11,12,13,14]. Until recently, it was thought that ERα is the predominant estrogen receptor in the H–P axis with ERβ having a negligible regulatory role on gonadotropin secretion. Using subfertile Erβ^KO^ female mice, it was shown that ERβ is not necessary within the H–P axis for generation of the gonadotropin surge [14]. This study emphasizes the presence of ERβ within the ovary for providing the required signals to the H–P axis, and suggests that estradiol alone may not be sufficient to induce the gonadotropin surge [14]. In contrast, a recent study has demonstrated that expression of ERβ in hypothalamic GnRH neurons is essential for induction of the preovulatory gonadotropin surge [30]. Moreover, loss of ERβ also reduces pulsatile GnRH production, and this mutation led to delayed onset of puberty in the Erβ^KO^ female mice [30]. 

Estrogen receptors also play an important role in the level of gonadotropin secretion from the pituitary gland [31,32]. ERα has been found essential for regulating LH and FSH secretion from the pituitary gonadotrophs, and thus female fertility [31]. It has been reported that ERβ can partially compensate the ERα deficiency in pituitary gonadotrophs [32]. Taken together, we can conclude that ERβ plays an important role in gonadotropin secretion from the H–P axis.

## 3. Ovarian Responses to Gonadotropins

**Figure 2:**
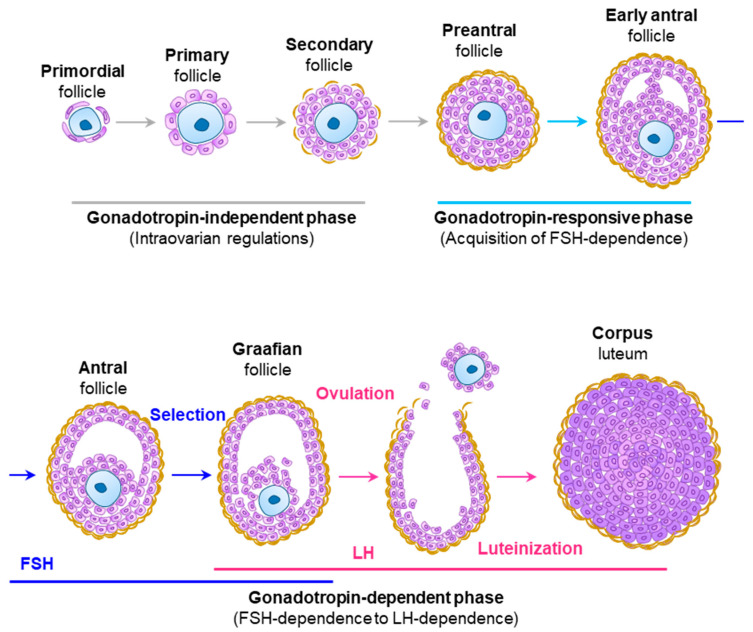
**A schematic representation of ovarian follicle development and ovulation.** At birth, a fixed number of primordial follicles are present in the ovary. Throughout a woman’s reproductive years, follicles are recruited and activated from the pool of dormant follicles. The initial recruitment of primordial follicles to form primary follicles, and their development into secondary follicles are regulated by intraovarian factors, which are independent of gonadotropins. When secondary follicles reach the preantral stage, developmental mechanisms of follicles shift from intraovarian to FSH responsiveness. Subsequent development of preantral follicles to early antral and then antral stage is FSH dependent. Thereafter, follicle selection is accomplished, follicles acquire LH-dependence and LH stimulation gives rise to the development of graafian follicles. LH-signaling is also crucial for the final stages of oocyte maturation, ovulation, and luteinization of GCs.

Gonadotropins play a vital role in ovarian development and onset of puberty [1]. Impaired gonadotropin secretion results in a something is missing here [30,33]. In the adult females, gonadotropins regulate the major ovarian functions: steroidogenesis and oogenesis [2,6]. Follicle assembly, activation of primordial follicles, and the early stage of follicle development to the preantral stage are independent of gonadotropins [2,34,35] (Figure 2). However, development of ovarian follicles beyond the early antral stage is dependent on FSH and LH stimulation [2,34,35]. The intraovarian regulators such as androgens, IGF1, EGF, activin, GDF9, BMP15, and connexins play vital roles in the acquisition of FSH dependence in preantral follicles [1]. Formation of the TC layer on secondary follicles is a key step for acquiring FSH dependence [1]. GC-derived KL and IGF1 recruits TCs to secondary follicles [36,37,38], and oocyte-derived GDF9 induces differentiation of the TCs [39,40,41,42]. These events are followed by expression of FSHR on GCs and LHCGR on TCs in preantral follicles [1]. TCs synthesize androgens that play important roles in the growth, survival, and acquisition of FSH dependence in preantral follicles [1]. Androgens bind to ARs in GCs to induce the expression of *Fshr*. IGF1 induces the expression of *Fshr* and *Cyp19a1* in GCs during the preantral-to-antral transition [43]. Expression of FSHR is highest in GCs of small antral follicles and the expression is decreased with further development and follicular selection [43,44]. In contrast, expression of LHCGR is increased in the GCs of larger antral follicles after selection and dominance [43,44]. IGF1, estradiol, and IL-6 can enhance the expression of *Lhcgr* gene that is induced by FSH stimulation [45,46,47]. While FSH-stimulation upregulates the expression of *Lhcgr* on GCs, LH-signaling downregulates it dramatically [45,46,47]. Limited information is available regarding the regulation of *Fshr* gene expression [48]. Activin and TGFβ can upregulate the expression of *Fshr*, but the mechanism remains unclear [48].

The development of early antral follicles to small antral follicles is dependent on FSH-induced follicular growth, whereas the development of antral follicles to the Graafian stage is mediated by LH-induced follicular (and oocyte) maturation [1,2,6] (Figure 2). Both growth and maturation phases of follicle development are accompanied by gonadotropin-induced steroidogenesis in TCs and GCs [1,2,6]. Pulsatile secretion of low levels of LH stimulates TCs to synthesize progestins, and androgens [49], which are taken up by the adjacent GCs and converted into estrogens [50,51]. A surge of gonadotropin secretion is triggered by the rising estrogen level synthesized by the GCs of maturing follicles. Preovulatory oocyte maturation, induction of ovulation, and luteinization of GCs are dependent on the gonadotropin surge.

LH and FSH have an identical α subunit, but the β subunit is different in each. This difference is responsible for the specific binding of each hormone to its cognate receptor [52]. However, the receptor binding is not exclusive of the β subunit because the α subunit also interacts with the gonadotropin receptors [52]. As we have mentioned above, only the somatic cells in ovarian follicles express the gonadotropin receptors. TCs express LHCGR and respond to LH stimulation, whereas mural GCs express both FSHR and LHCGR and respond to both gonadotropins [6,53,54]. As oocytes do not express gonadotropin receptors, the gonadotropin response from TCs or GCs is conveyed to them through vectorial transfer of information [6]. FSHR and LHCGR are G-protein coupled receptors (GPCRs) that activate adenyl cyclase, PKA, PI3K-AKT, and MEK1-ERK1/2 pathways. Gonadotropin responses can also be grouped into cAMP-dependent and cAMP-independent. Although both gonadotropins are thought to activate similar protein kinase pathways, the fundamental difference between FSH and LH response in the ovary results from cell-type specific expression of their receptors, and the dynamic differences in their pulsatile and bolus secretion from the anterior pituitary gland. 

### 3.1. FSH Signaling in the Ovary

FSHR is expressed in the GCs of multilayered secondary follicles, however, FSH stimulation is essential for follicle development beyond the preantral stage [2,34,35,55] (Figure 2). Secondary follicles acquire FSH dependence during the transition from preantral to early antral stage and these changes determine the fate of follicles [2,34,35]. In Fshβ^KO^ mice, activation of primordial follicles and subsequent growth to preantral follicles was intact, but follicles were arrested at the preantral stage, and no antral follicles were observed [34,56]. These findings indicate that FSH is indispensable for follicle growth and antrum formation during the preantral-to-antral stage transition [34,56].

**Figure 3:**
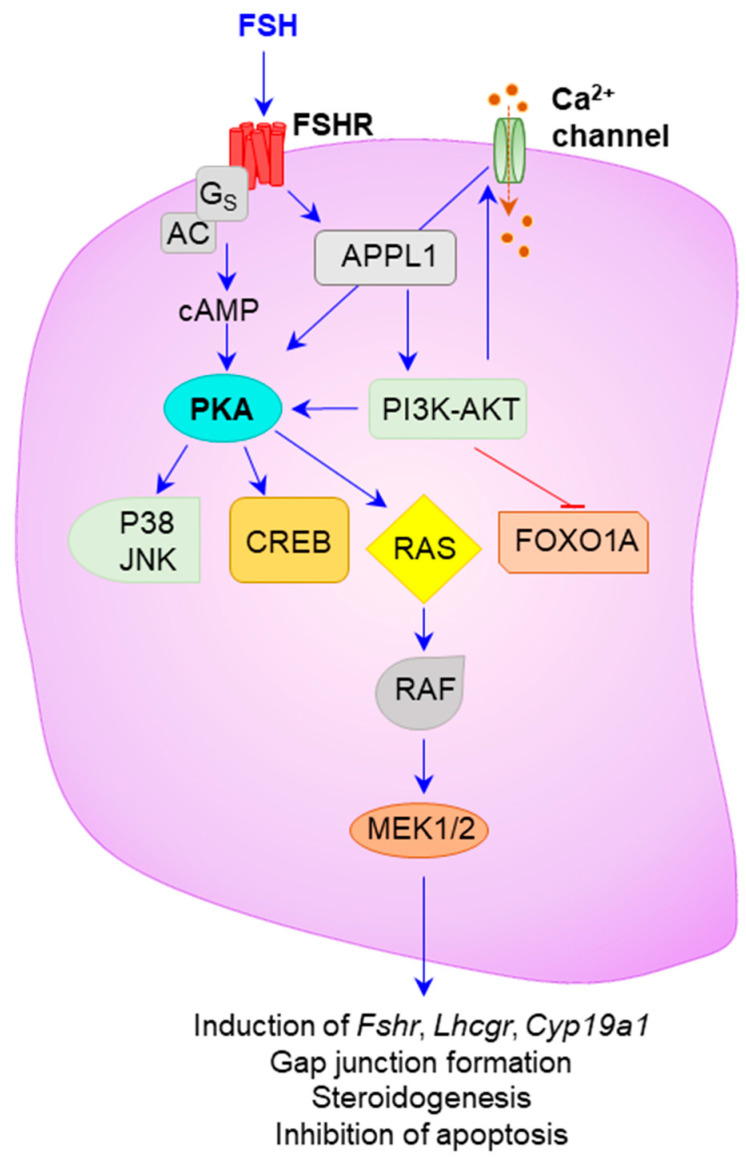
**FSH signaling in the ovarian follicles.** FSH signaling is necessary for the development of follicles during preantral to antral transition. Binding of FSH to FSHR can activate GCs in both a cAMP-dependent and independent manner. Upon FSH binding, FSHR recruits G_s_ and AC, leading to activation of the cyclic AMP/protein kinase A (cAMP/PKA) pathway. Alternatively, PI3K/AKT can be activated upon FSHR interaction with APPL1. Through phosphorylation, PI3K/AKT directly inhibits FOXO1A, which leads to upregulation of FOXO-regulated genes involved in cell proliferation. In addition, PI3K/AKT activation of Ca^2+^ channel leads to an increase in intracellular calcium concentration, which is crucial for follicle selection and dominance. PI3K/AKT can also activate the RAS/RAF/MEK singling that plays an important role in the induction of *Fshr*, *Lhcgr*, *Cyp19a1* expression, gap junction formation, steroidogenesis, and inhibition of apoptosis.

FSH activates the GCs both in a cAMP-dependent and a cAMP-independent manner [55,57]. Binding of FSH to FSHR activates adenyl cyclase and increases cAMP levels, which subsequently activates the PKA pathway [55,57] (Figure 3). FSH signaling can activate GRKs and associate with β-arrestins, which results in GPCR desensitization and G-protein independent signaling [58,59,60,61]. FSHR interacts with APPL1, and activates the PI3K-AKT and calcium ion mobilization essential for follicle selection and acquisition of dominance [62,63]. Activated FSHR can interact with the adapter protein 14-3-3τ, which can also mediate AKT-activation [64,65]. Activated PI3K-AKT phosphorylates and deactivates FOXO1A [66,67] that leads to upregulation of GC-genes involved in cellular proliferation [68]. FSH induced PI3K-AKT activation also inhibits apoptosis of GCs in antral follicles and prevents follicle atresia [2]. FSHR can interact with a PDZ protein, GIPC, that promotes the intracellular MAPK [69]. FSH signaling can also activate MEK1 and ERK1/2 by stimulating RAS–RAF–MEK pathway [62] (Figure 3). FSH can also stimulate the TGFβ pathway and activate transcription factors like SMAD2/3 and SMAD4 [70]. Thus, FSH signaling regulates the expression of target genes including *Lhcgr,* steroidogenic enzymes, protein kinases, and growth factors that positively impact steroidogenesis and gametogenesis [71,72,73,74,75,76,77,78] (Figure 3). Recent studies suggest that estrogen signaling increases the ovarian responses to FSH. Particularly, estradiol augments the FSH effects during the advanced stages of follicle development [79,80]. 

### 3.2. LH Signaling in the Ovary

Development of antral follicles to the Graafian stage occurs after follicle selection and dominance via LH-dependent mechanisms that increase estrogen synthesis and activate IGF1 signaling [1,2,81,82,83,84,85,86]. In antral follicles, LHCGR is expressed in both TCs, and mural GCs but not in cumulus GCs or oocytes [6]. FSH signaling in association with the intraovarian factors like IGF1, IL6 and estradiol induces *Lhcgr* expression in mural GCs [48], whereas it is repressed in cumulus GCs by GDF9 secreted from oocytes [87,88,89]. Lhβ^KO^ mice suffer from arrested antral follicle growth, and fail to develop preovulatory follicles, indicating that LH signaling is essential for further maturation of antral follicles [90,91,92]. 

**Figure 4:**
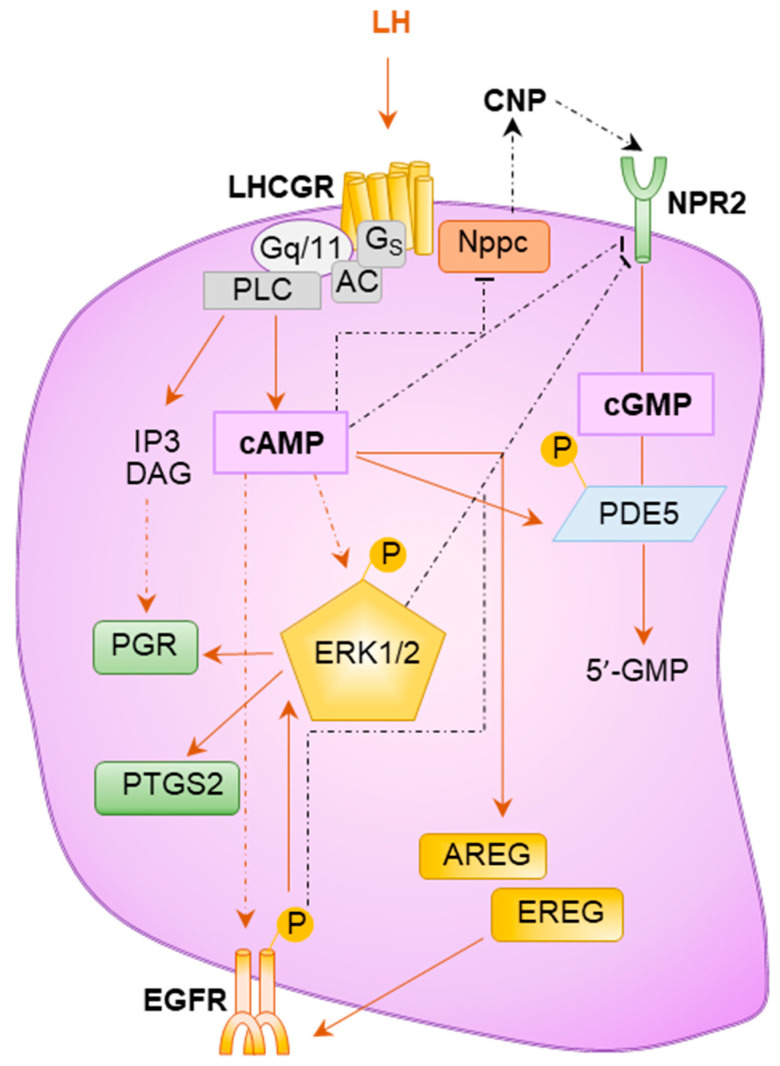
**LH signaling in the ovarian follicles.** The final stages of follicle maturation and ovulation are dependent on binding of LH to the LHCGR in mural granulosa cells (GCs). The binding of LH to the LHCGR activates Gs, which increases cAMP levels within mural GCs. LH stimulated GCs express growth factors including AREG and EREG that can stimulate the EGFR signaling. This results in an activation of RAS–RAF–MEK pathways that phosphorylate ERK1/2. Activated pERK1/2 stimulates the expression of *Pgr* and *Ptgs2*, which are necessary to achieve successful ovulation. In contrast, cAMP and ERK1/2 pathways inhibit expression *Nppc* mRNA (that encodes CNP) and NPR2, respectively. As CNP and NPR2 plays an important role in the maintenance of meiotic arrest in preovulatory follicles, the inhibition of CNP/NPR2 signaling allows oocytes to resume meiosis.

LH signaling in TCs plays an essential role in initiating steroidogenesis, whereas LH binding to LHCGR induces differentiation of GCs, which is required for cumulus expansion, oocyte maturation, ovulation, and luteinization [6,93]. The low level of LH bound to LHCGR readily activates G_s_ and stimulates cAMP synthesis. However, in the presence of a large quantity of LH and higher LHCGR expression during the preovulatory period, LH signaling can also activate G_q/11_, stimulate phospholipase C, and increase second messengers like inositol phosphates, calcium, and diacylglycerol [94,95,96,97] (Figure 4). 

LH signaling via LHCGR interacts with an RTK family member, EGFR, and a guanylyl cyclase NPR2^6^. LH stimulated mural GCs express EGFR ligands EREG, AREG, and others, which can activate EGFR [98,99,100,101] (Figure 4). These factors trigger RAS–RAF–MAPK pathways, and increase the expression of *Ptgs2*, *Has2*, and *Tnfaip6* in GCs, which are essential for the induction of ovulation [98]. In mutant mouse studies, disruption of the EGF pathway [102] or ERK1/2 [103] resulted in failure of ovulation despite a normal follicle growth. Thus, ERK1/2 may mediate the response of EGFR signaling in activated GCs [104]. LH stimulated mural GCs also express high levels of *Nppc* mRNA that encodes C-type natriuretic peptide ligand (CNP), which can activate NPR2 to increase the cGMP production crucial for follicle maturation [105,106].

### 3.3. Interaction between FSH and LH Signaling

FSHR can interact with other related GPCRs like LHCGR, and thus provide diversity in regulation of gonadotropin responses [107,108,109]. Studies have suggested that heteromerization of the FSHR with LHCGR plays a key role in regulating the follicular growth and selection [110,111]. Intracellular signals delivered by LHCGR may be modulated by the presence of FSHR on GCs, and vice versa. While unliganded FSHR can amplify LHCGR signals, LHCGR can inhibit FSHR-dependent cAMP production [110,112]. FSHR also interacts with RTKs including IGF1R and EGFR, which is important for the AKT and ERK1/2 activation required for gonadotropin induced differentiation of GCs [62,113,114]. 

## 4. ERβ Regulation of the Gonadotropin Responses 

For successful ovulation, ovarian follicles need to develop to full maturity in response to gonadotropin stimulation that leads to follicle rupture [100]. Estrogen signaling plays a crucial role in mediating an effective gonadotropin response on the ovarian follicles [10,100]. Thus, disruption of estrogen signaling by loss of estrogen receptors or aromatase prevents antral follicles from developing to the Graafian stage and to ovulate [10,13,115,116,117,118,119]. The expression and function of ERα are predominant at the H–P level, and that of ERβ are prominent within the ovary. Thus, ERα is important for gonadotropin secretion whereas ERβ is essential for gonadotropin responses in the ovary [10]. Nevertheless, ERβ also regulates gonadotropin secretion acting in GnRH neurons [30] and ERα also regulates steroidogenesis acting in TCs. 

An effective interaction between estrogen signaling and gonadotropin responses is required for the ovarian follicle maturation and ovulation. As the somatic cells express the gonadotropin receptors, it is likely that gonadotropin signaling interacts with the estrogen signaling within these cells. Loss of either ERα in TCs or loss of ERβ in GCs affects the gonadotropin responses regulating ovarian functions [120]. Somatic cells are primarily involved in steroidogenesis and regulation of oocyte maturation in response to gonadotropins [53,54]. While LH signaling initiates steroidogenesis in TCs, both FSH and LH signaling complete the final steps of steroidogenesis in GCs [53,54,121,122]. Further, LH stimulated GCs contribute to oocyte maturation, induction of ovulation, and formation of the corpus luteum [53,54,121,122].

**Figure 5:**
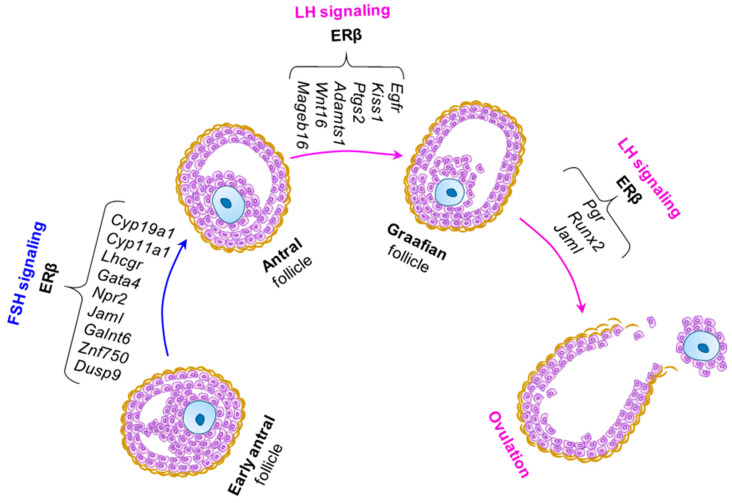
**ERβ regulation of gonadotropin responses.** ERβ is the predominant estrogen receptor in the ovary involved in transcriptional regulation of gene expression. While ERα is expressed in theca cells (TCs), ERβ is expressed in granulosa cells (GCs). As GCs express both FSHR and LHCGR, we analyzed the role of ERβ in gonadotropin-induced gene expression in GCs. We identified that a subset of PMSG (that activates FSHR) or hCG (that activates LHCGR) regulated genes failed to respond in the absence of ERβ expression in GCs. In early antral follicles, expression of FSHR-induced genes including *Cyp19A1*, *Cyp11a1*, *Lhcgr*, *Gata4*, *Npr2*, *Jaml*, *Galnt6*, *Znf750*, and *Dusp9* was dependent on ERβ. Moreover, presence of ERβ was found to be essential for the expression of LHCGR-induced genes, such as *Egfr*, *Kiss1*, *Ptgs2*, *Adamts1*, *Wnt16*, *Mageb16*, *Pgr*, *Runx2*, and *Jaml*. Disruption of ERβ signaling results in dysregulation of these genes and is associated with failure of follicle maturation, and ovulation. As ovulation does not occur in the absence of ERβ, the potential role of ERβ in luteinization has not been studied.

Gonadotropin responses in the ovary are affected in the absence of ERβ [10,100,101]. Erβ^KO^ mutant female mice and rats have been found to be infertile due to failure of follicle maturation and ovulation [10,100,101]. However, loss of ERβ does not affect the male reproductive function [10]. Targeted deletion of the DNA-binding-domain of ERβ resulted in an anovulatory phenotype in mutant rats similar to that of complete Erβ^KO^ rats, suggesting that canonical transcriptional regulatory function of ERβ is essential for the gonadotropin responses [10,100]. Due to a high level of ERβ expression in GCs, ERβ-regulated GC-genes play crucial roles in folliculogenesis starting from follicle assembly and follicle activation to follicle maturation and ovulation [100,101]. Presence of ERβ is essential for the gonadotropin-induced differentiation of GCs, and regulation of GC-genes including *Lhcgr* and the steroidogenic enzyme *Cyp19a1* as well as the transcriptional regulator *Pgr* [101,123] (Figure 5). Transcriptional regulators are either activated or inactivated by LH or FSH stimulation resulting in differential expression of genes in TCs or GCs that are required for steroidogenesis, follicle development, and oocyte maturation. One such group of transcriptional regulators are estrogen receptors within the somatic cells. However, instead of being a downstream target of gonadotropin signaling, estrogen receptors may also have gonadotropin-independent roles that are required for ovarian follicle development and oocyte maturation [7,124]. 

ERβ is a ligand-activated transcription factor. However, loss of ERβ disrupts the final stages of follicle development and oocyte maturation, when gene transcription is minimal in oocytes. Studies have shown that ERβ can induce the expression of miRNAs [125] and it can directly interact with AGO2 [125]. Thus, ERβ can also be involved in posttranscriptional regulation of gene expression. Nevertheless, most of the studies suggesting a post-transcriptional regulatory function of ERβ refer to cancer cells, and it remains unknown whether such mechanisms also occur in normal ovarian follicles. 

### 4.1. ERβ Regulation of FSH Responses

FSH signaling stimulates early antral follicles to develop to the antral stage [2]. It has been shown that FSH stimulation of small antral follicles alone is insufficient for induction of maturation, which must be facilitated by estrogen signaling [126]. Loss of ERβ does not impact the development of ovarian follicles prior to the antral stage [101]. However, failure of Erβ^KO^ follicles to mature following LH stimulation suggests that those follicles may not possess the factors required for a proper LH response [101]. Gene expression analyses 48h after PMSG stimulation (PMSG acts on rodent FSHR) revealed that many of the genes that are differentially expressed in wildtype ovaries fail to do so in the absence of ERβ [10,100,101]. 

Most studies suggest a primary role for ERβ in the GCs as being essential for FSH induced ovarian follicle development. Differentiation of GCs in response to FSH is dependent on ERβ-mediated estrogen signaling [127]. Despite an increased expression of FSHR, administration of PMSG fails to induce the genes required for an effective LH response [101,128]. Although there was no change in FSH-induced genes such as *Star*, expression of *Lhcgr*, *Cyp11a1*, *Cyp19a1*, *Gata4*, and *Npr2* failed to upregulate in Erβ^KO^ GCs [10,100,101,129] (Figure 5). These findings suggest that expression of a subset of FSH-induced genes is dependent on the presence of ERβ in GCs [101].

In the absence of ERβ, FSH-induced cAMP production is markedly reduced in GCs [79]. However, the molecular mechanism underlying such reduced cAMP production in GCs remains unclear [79]. In vitro and in vivo studies have also demonstrated defective antrum formation, associated with decreased cumulus expansion after FSH treatment [120,129,130]. Due to the reduced levels of *Cyp11a1* and *Cyp19a1*, GCs in Erβ^KO^ preovulatory follicles exhibit significantly lower levels of FSH-induced estrogen synthesis [120,130]. A decreased level of *Cyp19a1* can interrupt the development of antral follicles to the Graafian stage. Similar to Erβ^KO^ mice, *Cyp19a1* knockout mice are able to develop antral follicles but failed to mature or ovulate [118]. Erβ^KO^ GCs also have a reduced level of *Gata-4* expression, which decreases the proliferation of GCs and that impairs follicle maturation [131,132]. In contrast, in vitro culture experiments with Erα^KO^ models detected a minimal role for ERα in the differentiation of GCs and their gene regulation [120,130].

### 4.2. ERβ Regulation of LH Responses

ERβ plays a very important role in the LH-induced differentiation of GCs required for follicle maturation and induction of ovulation [130]. A reduced level of *Lhcgr* expression in Erβ^KO^ GCs in response to FSH causes failure of those antral follicles to respond to LH, which is essential for their development to Graafian follicles [6,133]. Expression of LH target genes that regulate steroidogenesis, cumulus cell expansion, oocyte maturation, and ovulation, were markedly impaired in Erβ^KO^ ovaries due to the failure of *Lhcgr* upregulation in Erβ^KO^ GCs [14,101]. It is important to note that *Lhcgr* knockout mice also suffered from lack of follicle development beyond the antral stage and failed to form Graafian follicles [91]. We recently reported a similar ovarian phenotype in gonadotropin-induced Erβ^KO^ rats [101]. 

Our recent study revealed that a subset of LH-induced genes in GCs is also dependent on the presence of ERβ [100,101]. We observed that hCG-stimulation (hCG activates LHCGR) failed to upregulate the expression of *Pgr*, *Runx2*, *Egfr*, *Ptgs2*, *Adamts1,* and *Kiss1* in Erβ^KO^ GCs [101] (Figure 5). *Pgr*, *Runx2*, *Ptgs2,* and *Adamts1* were also found to be downregulated in GCs isolated from hCG treated Erβ^KO^ mice [123]. We previously demonstrated that loss of ERβ results in failure of LH-induced *Kiss1* gene expression in Erβ^KO^ rat GCs [100,101]. Our recent findings suggest that ERβ-regulated ovarian kisspeptin may play an important role in preovulatory maturation of oocytes [129]. However, it remains unknown if ovarian kisspeptin has any role in regulating GnRH neurons. In addition to the known LH-regulated genes, we identified that loss of ERβ also alters the expression of several novel GC-genes including *Jaml*, *Galnt6*, *Znf750*, and *Dusp9* [101]. Differential expression of these LH-regulated genes in GCs may be responsible for the lack of maturation, and ovulation of Erβ^KO^ ovarian follicles [101].

LH signaling also plays an important role in TCs, however, the major estrogen receptor in TCs is ERα. Therefore, it is less likely to be impacted by ERβ. However, development of the TC layer, and differentiation of TCs can be affected by the loss of ERβ in GCs or oocytes, because these mechanisms are dependent on GC-derived KL and IGF1 [36,37,38] and oocyte derived GDF9 [39,40,41,42]. We have observed that serum androstenedione and progesterone levels can be lower in Erβ mutant rats [100]. However, studies have not yet analyzed the changes in the gene expression profile in Erβ^KO^ TCs. 

## 5. Chorionic Gonadotropins in Ovarian Biology

Two placenta-derived gonadotropins (chorionic gonadotropins) are commonly used in ovarian biology research and in clinical settings. Human chorionic gonadotropin (hCG) is a polypeptide hormone produced by the trophoblast cells of the placenta. Equine chorionic gonadotropin (eCG), also known as pregnant mare serum gonadotropin (PMSG), is another commonly used placenta-derived gonadotropin hormone. Chorionic gonadotropins are composed of two dissimilar subunits of glycoproteins like that of pituitary gonadotropins. The α subunit is common to chorionic and pituitary gonadotropins while the β subunit, which is unique for each specific hormone, is responsible for selective receptor binding. The β subunit of hCG (β-hCG) has an 85% homology with the β subunit of pituitary LH, but in equids, the β subunit of chorionic gonadotropin and pituitary LH are expressed from the same gene, differing only by the glycosylation pattern. β-hCG is mostly similar to β-LH, differing in the carboxy terminal region. β-hCG has a carboxy terminal extension that includes four glycosylated serine residues that is responsible for its longer half-life. hCG can bind and activate LHCGR in humans as well as in experimental animals like rodents. Interestingly, PMSG has only LH-like activity in equids, but in other species including rodents, it has FSH-like activity due to its preferred binding to FSHR. PMSG is also preferred over pituitary extracts of gonadotropins due its longer half-life. hCG prepared from the urine of pregnant women and PMSG purified from pregnant horse serum are used in research, however, recombinant hCG or PMSG have been developed and approved for clinical use. 

Physiologically, CGs are important only during pregnancy in humans, primates, and horses [134,135]. These mammals sustain their initial period of pregnancy by steroid hormones produced by the corpora lutea. Extension of normal corpus luteum life is achieved by placental secretion of chorionic gonadotropins and their binding to and regulation of LHCGRs within the corpus luteum. Subsequently, they experience a luteal to placental shift, and placental steroid production becomes essential for continuing their pregnancy [134,135]. In contrast, the rodent corpora lutea are responsible for steroid hormone production throughout gestation. Therefore, the rodents do not express CGs in placenta to sustain their pregnancy [134,135]. In animal experiments, exogenous CGS (PMSG and hCG) are administered into mice or rats for synchronized induction of ovarian follicle development, as well as for the induction of ovulation. PMSG is administered to act like FSH while hCG is administered to act like LH. hCG can bind the LHCGR and induce responses like that of LH signaling. Injections of hCG mimic the LH surge that is necessary for oocyte maturation and induction of ovulation. hCG is also used in the therapy of female infertility, particularly in assisted reproductive techniques. PMSG is also administered with progesterone to induce ovulation in livestock prior to artificial insemination. 

Another importance of CG is the potential role of hCG in cancer progression due to its proangiogenic properties [136]. Ovarian cancer cells express hCG and its receptor LHCGR [137]. Such aberrant expression of hCG can be used as a tumor marker in nonpregnant females [138,139]. It has been shown that hCG stimulates angiogenesis in the ovary by inducing the expression of VEGF and increasing the proliferation of vascular endothelial cells [137,140]. However, there has been no correlation between hCG expression and the survival of ovarian cancer patients [141]. An interesting aspect of LHCGR expression outside the H–P–O axis is the association and sensitivity of the expression site with estrogen signaling [137,140]. Tissues that express LHCGR also respond to changes in estrogen levels [142], which suggest that either estrogen can modulate the expression of LHCGR or estrogen signaling interacts with LH signaling. Thus, cancer cells that express LHCGR may also express ERα and ERβ and respond to estrogen signaling. However, further studies are required to clarify that. 

## 6. ERβ and Gonadotropins in Ovarian Diseases

In contrast, hCG acts on increasing the growth and angiogenesis of ovarian cancers as mentioned above. However, it remains unclear how gonadotropin signaling and ERβ signaling interact in ovarian cancer cells. ERβ is the predominant estrogen receptor in the ovary [143,144,145,146]. ERβ polymorphisms and mutations in women have been linked to ovulatory dysfunctions, including complete ovarian failure [147,148,149,150]. PCOS, a common clinical condition among women that causes failure of ovulation and infertility, is associated with high levels of LH and androgens [151,152]. Recent genomewide association studies have linked FSH and LH receptor variants to the development of PCOS [153]. Due to the intricate connection between gonadotropin response and estrogen signaling in the ovary, it is likely that estrogen signaling plays an important role in the pathogenesis of PCOS. The loss of ERα induces polycystic like changes in mutant mouse [154] and rat [115] ovaries. But there are no such cystic changes in the Erβ^KO^ mouse [12,13] or rat [10] ovaries. Rather, the presence of ERβ was found essential for the development of polycystic changes in Erα^KO^ mice [146]. Based on these findings, it may be assumed that loss of ERα in TCs associated with a normal or increased ERβ activity in GCs may lead to the development of PCOS. However, studies on human PCOS tissues only partially support the assumption [155,156,157,158]. Another ovarian disease that has been linked to estrogen signaling is ovarian cancer [159]. Estrogen receptors are also frequently detected in ovarian cancers, however the exact role of estrogen receptors in ovarian cancer prognosis remains unclear [159,160,161,162,163]. ERβ acts as a tumor suppressor and inhibits the progression of ovarian cancers [164,165]. As expected, expression of ERβ is very low in advanced ovarian cancers [166,167] and loss of ERβ expression in ovarian cancers correlates with a shorter survival rate [168,169]. In contrast, hCG acts on increasing the growth and angiogenesis of ovarian cancers as mentioned above. However, it remains unclear how gonadotropin signaling and ERβ signaling interact in ovarian cancer cells. 

## 7. Future Perspectives

Estrogen signaling is essential for mediating effective gonadotropin responses within the ovary. Gonadotropin receptors are expressed in TCs and GCs. The presence of ERα in TCs, and ERβ in GCs are essential for gonadotropin induced steroidogenesis and gametogenesis. However, it remains unclear how gonadotropin signaling interacts with estrogen signaling, and the hierarchy in these signaling mechanisms in those somatic cells. It has been demonstrated that FSH induced *Lhcgr* expression in GCs depends on the presence of ERβ [100,101]. As loss of ERβ reduces estrogen synthesis in GCs, it may be hypothesized that ERβ-dependent estrogen signaling positively regulates *Lhcgr* gene expression in GCs. In contrast, the expression of *Fshr* is increased in the absence of ERβ in the ovary [100,101], which suggest that ERβ may negatively regulate *Fshr* expression in GCs. Nevertheless, the molecular mechanisms underlying ERβ regulation of gonadotropin receptors in GCs remain unknown.

ERβ is the predominant estrogen receptor in the ovary, where it functions to regulate expression of genes involved in follicle development and oocyte maturation [120,170,171,172]. GCs in growing ovarian follicles express the highest level of ERβ. However, in vitro studies on GCs are limited by spontaneous differentiation of GCs in culture. Moreover, GCs rapidly lose the expression of ERβ in cell culture. Thus, the results obtained from in vitro studies of GCs may differ from the exact molecular mechanisms that exist in vivo. Another limitation in ERβ research is the lack of a specific antibody [173]. Although a mouse monoclonal antibody has been reported to be efficient in detecting human ERβ, it fails to detect ERβ in the rodents [173].

Our studies have shown that ERβ plays a major role in regulating the GC-genes that are important for oocyte maturation and induction of ovulation [10,100,101,129]. Administration of gonadotropins for ovarian stimulation is a common practice in assisted reproductive technologies [174,175]. Some of the patients that receive gonadotropins do not respond well and are investigated for predisposing conditions underlying the defective gonadotropin responses [174]. A more directed focus on ERβ may help identify the underlying pathologies and lead to an effective treatment to overcome ineffective follicle development and oocyte maturation following gonadotropin stimulation. 

## Data Availability

SRA PRJNA551764 and PRJNA551766.
